# Pulsed Electromagnetic Field Regulates MicroRNA 21 Expression to Activate TGF-*β* Signaling in Human Bone Marrow Stromal Cells to Enhance Osteoblast Differentiation

**DOI:** 10.1155/2017/2450327

**Published:** 2017-04-23

**Authors:** Nagarajan Selvamurugan, Zhiming He, Daniel Rifkin, Branka Dabovic, Nicola C. Partridge

**Affiliations:** ^1^Department of Biotechnology, School of Bioengineering, SRM University, Kattankulathur, Tamil Nadu, India; ^2^Department of Basic Science and Craniofacial Biology, New York University College of Dentistry, New York, NY, USA; ^3^Department of Cell Biology, New York University School of Medicine, New York, NY, USA

## Abstract

Pulsed electromagnetic fields (PEMFs) have been documented to promote bone fracture healing in nonunions and increase lumbar spinal fusion rates. However, the molecular mechanisms by which PEMF stimulates differentiation of human bone marrow stromal cells (hBMSCs) into osteoblasts are not well understood. In this study the PEMF effects on hBMSCs were studied by microarray analysis. PEMF stimulation of hBMSCs' cell numbers mainly affected genes of cell cycle regulation, cell structure, and growth receptors or kinase pathways. In the differentiation and mineralization stages, PEMF regulated preosteoblast gene expression and notably, the transforming growth factor-beta (TGF-*β*) signaling pathway and microRNA 21 (miR21) were most highly regulated. PEMF stimulated activation of Smad2 and miR21-5p expression in differentiated osteoblasts, and TGF-*β* signaling was essential for PEMF stimulation of alkaline phosphatase mRNA expression. Smad7, an antagonist of the TGF-*β* signaling pathway, was found to be miR21-5p's putative target gene and PEMF caused a decrease in Smad7 expression. Expression of Runx2 was increased by PEMF treatment and the miR21-5p inhibitor prevented the PEMF stimulation of Runx2 expression in differentiating cells. Thus, PEMF could mediate its effects on bone metabolism by activation of the TGF-*β* signaling pathway and stimulation of expression of miR21-5p in hBMSCs.

## 1. Introduction

Abundant reports describe the effects of electricity on bone growth and fracture repair, and a variety of pulsed electromagnetic field (PEMF) devices have been developed to produce electromagnetic fields at the fracture site. These widespread PEMF devices utilize noninvasive inductive coupling and can be used along with every method of fracture fixation [1, 2]. The stimulation of bone at the fracture site by the introduction of electromagnetic fields may be similar to the resulting stimulation from mechanical loading [1]. The beneficial therapeutic effects of such selected low energy, time varying PEMF promote fracture healing in nonunions [3], increase lumbar spinal fusion rates [4, 5], and have been found to affect bone metabolism by decreasing bone resorption and increasing bone formation [6–8]. PEMFs have also been reported to stimulate the synthesis of extracellular matrix (ECM) proteins [9] and may also affect several membrane receptors including those for parathyroid hormone, low density lipoprotein, insulin-like growth factor-2, insulin, and calcitonin [10]. Several growth factors such as bone morphogenetic proteins 2 and 4 (BMP-2, BMP-4) and transforming growth factor-beta (TGF-*β*) have been reported to be secreted from osteoblasts upon PEMF treatment [11]. It has been shown that electromagnetic stimulation could raise net Ca^2+^ flux in human osteoblast-like cells, and the increase in the cytosolic Ca^2+^ concentration could initiate activation of signaling pathways resulting in regulation of expression of bone matrix genes [12, 13]. Accelerated osteogenesis has been found in bone marrow-derived mesenchymal stem cells by PEMF treatment [14] and this promotion of ECM deposition was more efficient compared with adipose-tissue mesenchymal stem cells [15].

Previously we have reported that both BMP-2 and PEMF (Spinal-Stim® by Orthofix, Inc., Lewisville, TX) separately stimulated proliferation of rat primary calvarial osteoblastic cells and stimulated expression of early osteoblast differentiation genes in culture [7]. In this study, we investigated the effects of PEMF (Cervical-Stim® by Orthofix, Inc., Lewisville, TX) on human bone marrow stromal cells (hBMSCs) proliferating and differentiated to osteoblastic cells. In addition, the underlying molecular mechanisms by which PEMF stimulates differentiation of hBMSCs into osteoblasts are not well understood. Thus, we also aimed to investigate the PEMF effects on proliferation, differentiation, and mineralization of hBMSCs by Affymetrix microarray analysis. The TGF-*β* signaling pathway and microRNA 21 (miR21) were most highly regulated by PEMF. Thus, in this study we systematically investigated the mechanism of action of PEMF effects on osteogenesis via TGF-*β* and miR21 using hBMSCs.

## 2. Materials and Methods

### 2.1. Cell Culture

Fresh human bone marrows from 21–68-year-old women were used. These were either purchased from Lonza (Walkersville, MD) or left over tissue from surgical procedures at New York University Hospital for Joint Diseases. Since these were deidentified, this is not considered Human Subjects Research by the New York University School of Medicine Institutional Review Board. In both cases, the bone marrows were freshly collected, never frozen, and immediately diluted 1 : 1 in Hank's Balanced Salt Solution (HBSS; GIBCO Laboratories, Grand Island, NY) containing 20 IU/mL of sodium heparin (Sigma Chemical Co., St. Louis, MO). The diluted bone marrow was layered over an equal volume of Ficoll-Paque Plus (GE Healthcare, Piscataway, NJ) and centrifuged at 400*g* for 40 min at 18°C. The mononuclear cells at the interface layer were collected, washed three times with HBSS, resuspended and seeded into a tissue culture flask, and incubated at 37°C in the presence of 5% CO_2_ overnight. The next day, nonadherent cells were removed from the culture flask. Adherent cells (BMSCs) were grown to confluence then placed in 6-well plates at 6.4 × 10^4^ cells/well for exposure to PEMF or control. All cells were incubated at 37°C in the presence of 5% CO_2_. The medium used for culturing these cells was *α*-MEM (Corning, Tewksbury, MA) containing 15% fetal bovine serum (FBS; GIBCO, Grand Island, NY) and Penicillin-Streptomycin (GIBCO, Grand Island, NY).

### 2.2. PEMF Exposure

The PEMF was generated as previously described [7] but was set to have similar waveform characteristics to a commercial, clinically approved proprietary device (Cervical-Stim by Orthofix Inc., Lewisville, TX). Cervical-Stim is the only device approved by the FDA for cervical fusion use and has been reported to be safe and effective [16]. The specific differences from our previous publication [7] were a burst frequency of 15 Hz and a burst period of 67 ms. The induced magnetic field was vertical relative to the surface of the plates. The PEMF waveform was routinely checked for its consistency using a field probe and oscilloscope. The first PEMF exposure was initiated 24 h after seeding cells in wells (day 1) and continued through the entire experiment. Control plates were placed in an identical incubator on Plexiglas shelves. The CO_2_ concentration, humidity, and temperature of the control and treatment incubators (upper and lower chambers of the same double incubator) were identical and were not affected by the PEMF.

### 2.3. Cell Number

Cells were grown in normal growth medium and were trypsinized, resuspended, and counted using a hemocytometer when they reached 70–80% confluence on day 10 or 20 of culture, respectively, for the BMSCs from the younger (21–30) women versus those from the 31–65-year-old women.

### 2.4. Osteoblast Differentiation

Human BMSCs were seeded at 6.4 × 10^4^ cells/well in 6-well cell culture plates and cultured for 10 days or 20 days in normal cell culture medium (*α*MEM + 15% FBS + 1% Penn/Strep) before they reached confluence. They were then cultured for an additional 13 (differentiation) or 23 (mineralization) days in osteogenic medium [normal growth medium supplemented with 10^−4 ^M L-ascorbic acid, 10^−8 ^M dexamethasone, and 1.80 mM potassium phosphate monobasic (Sigma, St. Louis, MO)]. The medium was changed three times/week.

### 2.5. Von Kossa Staining

For Von Kossa staining, 6 replicates of BMSCs were treated with PEMF or control daily from day 1 of culture. On day 23, 33, or 43, the cells were fixed with 95% ethanol for 15 min at 37°C, then rinsed and rehydrated through 80%, 50%, and 20% ethanol and then water, and incubated with 5% silver nitrate solution for 1 h at 37°C. The cells were rinsed with water, exposed to UV light for 10 min, and photographed. Von Kossa staining was analyzed by computer based morphometry (ImageJ: NIH, Bethesda, Maryland).

### 2.6. Extracellular Regulated Kinases Activation and Western Blot Analyses

Human BMSCs treated with control or PEMF for 5 and 10 days in the proliferation phase were washed with cold phosphate buffered saline (PBS) and lysed in Cell Lysis Buffer (Invitrogen, Grand Island, NY) containing protease and phosphatase inhibitor cocktails (Sigma). Cell lysates were centrifuged at 10,000 rpm for 10 minutes at 4°C and supernatants were saved and used for Western blot analysis. Twenty *μ*g of total cell protein was loaded per well and separated on 4–15% Mini-Protean TGX precast gels (Bio-Rad, Hercules, CA), followed by transferring to nitrocellulose membranes (Bio-Rad, Hercules, CA). The membranes were blocked and incubated with primary rabbit antibodies (Phospho-p44/42 MAPK (Erk1/2) (Thr202/Tyr204), p44/42 MAPK (Erk1/2) (137F5), or Cdk2 (sc-163; Cyclin dependent kinase 2, loading control)) overnight at 4°C. The membranes were then probed with secondary antibody conjugated with horseradish peroxidase. Finally, the bands were visualized by adding Super Signal West Dura Extended Duration Substrate (Thermo Scientific, Pittsburgh, PA) according to the manufacturer's instructions. The primary antibodies to total ERKs and phosphorylated ERKs were obtained from Cell Signaling Technology (Danvers, MA), while the antibody to Cdk2 was obtained from Santa Cruz Biotechnology, Inc. (Dallas, TX). The secondary antibody (goat-anti-rabbit) conjugated with horseradish peroxidase (HRP) was obtained from Santa Cruz Biotechnology. Results were captured and quantitated by ChemiDoc XRS+ software (Bio-Rad, Hercules, CA). Both the Phospho-ERK1/2 and total ERK 1/2 were normalized to Cdk2 and then expressed as a percent of the values obtained in untreated control cells.

### 2.7. Microarray Assays

Human BMSCs of a 27-year-old healthy female donor were used for microarray experiments. Only hBMSCs expanded from the second to sixth passages were used for the experiments. PEMF treatment (Cervical-Stim) was initiated 24 h after hBMSCs were seeded, with 4 h daily exposure every day throughout the experimental period. Quadruplicate cell samples from both PEMF-treated and control groups were collected simultaneously at time points of hBMSC proliferation, osteoblast differentiation, and mineralization phases. Total RNA was isolated from cells by using TRIzol reagent (Thermo Scientific, Pittsburgh, PA) and then purified with RNeasy mini kit from Qiagen (Valencia, CA). Prior to microarray analysis, the RNA integrity was assessed by Agilent 2100 Bioanalyzer (Santa Clara, CA) and the best quality triplicate samples were chosen for the subsequent analyses. Microarrays and data analyses with Affymetrix Human U133 plus 2.0 Gene Chips (Santa Clara, CA) were performed at University of Medicine and Dentistry of New Jersey Genome Center according to the manufacturer's instructions. In the case of gene expression where it was significantly found to be above 1.5-fold after PEMF treatment, gene ontology analysis was carried out by DAVID Bioinformatics Resources 6.7 software (NIAID, NIH).

### 2.8. Real-Time RT-PCR

Total RNA was isolated from cells using the total RNA isolation kit from Qiagen (Valencia, CA). For determination of expression of genes other than miR21-5p, 100 ng of total RNA from each sample was used for cDNA synthesis using TaqMan Reverse Transcription Reagents (Roche, Indianapolis, IN). Quantitative (q)PCR reactions were performed according to the real-time thermocycler machine (Realplex) manufacturer's instructions (Eppendorf, Hauppauge, NY), which allowed real-time quantitative detection of the PCR products by measuring the increase in SYBR green fluorescence caused by binding of SYBR green to double-stranded DNA. The Power SYBR green master mix kit for PCR reactions was purchased from Invitrogen. The qPCR was performed in triplicate with reaction conditions of 95°C, 10 min, 1 cycle; 95°C, 15 sec; and 58.5°C, 1 min, for 40 cycles. Gene expression was analyzed with threshold cycle (CT) values averaged from triplicate samples and normalized to their CT values of housekeeping gene RPL13A. Primers were designed by NCBI primer Blast software.  Table 1 lists the human-specific primers used for PCR amplification.

For miR21-5p and snoR10-1, the reagents and primer sets for RT-qPCR were purchased from Qiagen. One ug of total RNA was reverse-transcribed into cDNA using the miScript II kit with miScript HiSpec Buffer according to the manufacturer's instructions. The cDNA was then diluted 10 times and utilized as a template to amplify miR21-5p and snoR10-1 with the miScript SYBR Green PCR kit using the appropriate primers. snoR10-1 was used as normalizing gene control. The qPCR was performed in triplicate with reaction conditions of 95°C 15 min for Taq DNA polymerase activation, 94°C 15 sec denaturation, 55°C 30 sec annealing, and 70°C 30 sec extension for 40 cycles. Gene expression results of miR21-5p from either control or PEMF-treated groups were normalized to their relative snoR10-1 results.

### 2.9. TGF-*β* Signaling

Human BMSCs were cultured and treated with control or PEMF as described above. For TGF-*β* and BMP signaling assays, osteoblasts were treated with PEMF at days 23 and 33 and were also treated with TGF-*β*2 (R&D System, Minneapolis, MN) as a positive control for the TGF-*β* pathway. The day before assay, the cells were starved overnight (0.1% FBS medium) to reduce endogenous signaling activity. At day 23 at the same time as PEMF exposure started, 5 ng/mL TGF-*β*2 was added to the medium of positive control wells. Cell lysates from different groups were collected at 0, 2, and 4 h time points after treatment to examine Smad2, Smad3, and Smad1/5/8 protein phosphorylation by Western blot analysis as described above. Phospho-Smad2 (Ser465/467, 138D4)/Smad2 (D43B4), phospho-Smad3 (Ser423/425, C25A9)/Smad3 (C67H9), and phospho-Smad1/5/8/Smad1/5 antibodies were obtained from Cell Signaling Technology (Danvers, MA). In TGF-*β* neutralization experiments, 30 ug/mL normal rabbit IgG or TGF-*β* pan antibody (R&D System, Minneapolis, MN) was added to osteogenic medium during the entire differentiation period. At day 23, two non-PEMF-treated cell groups were also included with 5 ng/mL of TGF-*β*2 as positive controls. After 2 h of PEMF exposure, all sample groups were collected for Western blots and RT-qPCR assays.

### 2.10. Transient Transfection

Cells were seeded in growth medium in 6-well plates at a density of 10^5^ cells/well on the day before the transfection. miR-21 is now referred as miR-21-5p, based on the latest miRBase release (V.21). miR21-5p inhibitor (Applied Biosystems: 4464084) designed to bind with endogenous miR21, when introduced into cells, inhibits its activities. miR21-5p mimic (Applied Biosystems: 4464066) was designed to be similar to that of endogenous miR21. A negative control miRNA (Applied Biosystems: 4464076) was included in the study. The X-treme Gene transfection reagent obtained from Roche, USA, was mixed with 50 nM of negative control miRNA, miR21 mimic, or miR21 inhibitor, and transient transfection was carried out [17] for 3 or 6 days along with PEMF treatment every day for 4 h.

### 2.11. Statistical Analysis

Statistical analysis was done by one-way ANOVA, Student's *t*-test, or Wilcoxon Ranking. Significant difference is *p* < 0.05. All data are shown as mean ± standard deviation with *n* as indicated.

## 3. Results

### 3.1. PEMF Effects on Proliferation and Differentiation of Human BMSCs from Subjects of Different Ages

**Figure 1:**
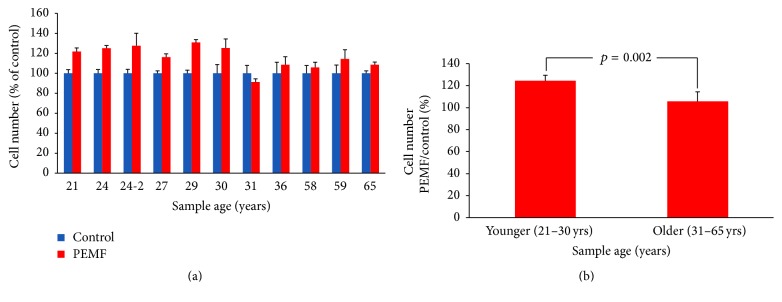
Effect of PEMF on hBMSC preosteoblastic cell number. (a) Human preosteoblasts derived from bone marrow stromal cells of 21–36-year-old women were treated with PEMF for 10 days; cells from 58-, 59-, and 65-year-old women were treated with PEMF for 20 days. Cell number/well was calculated using a hemocytometer (*n* = 3–6 wells). (b) Aggregation of the data into the two age groups, *n* = 5-6. The statistical *p* value for the younger versus older samples is shown using Student's *t*-test analysis.

**Figure 2:**
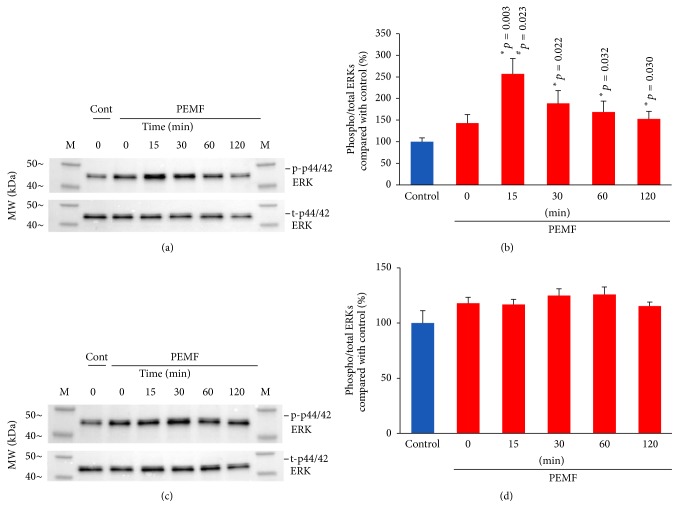
Effect of PEMF on ERK activation. Human BMSCs from two different 24-year-old women and a 27-year-old woman were subjected to 4 h daily PEMF treatment for either 4 days or 9 days. On the 5th (a) or 10th day (c), their cells were treated with PEMF for different time periods as indicated and whole cell lysates were obtained and subjected to Western blot analyses; cells from a 24-year-old woman are shown as an example. ((b) day 5, (d) day 10) The quantitation of activated or phosphorylated ERKs for cells from 2 separate 24-year-old women and a 27-year-old woman was determined by normalization of phosphorylated ERKs to total ERKs after normalization to Cdk2 as a loading control and expressed as a percent of untreated control cells. The results are shown for the cells of the 3 individuals. *∗* indicates significant increase compared to control. # indicates significant increase compared to 0 times of PEMF on the 5th day. The* p* value ≤ 0.05 is considered as significant using one-way ANOVA.

We previously reported that PEMF generated by Spinal-Stim stimulated cell proliferation and expression of early differentiation marker genes in rat primary calvarial osteoblastic cultures [7]. In the present study we used PEMF (Cervical-Stim) to determine its effect on osteoblasts using human bone marrow cells. PEMF significantly stimulated the cell number of preosteoblasts from BMSCs of young women (21–30 years old) while not stimulating those of BMSCs from 31–65-year-old women (Figures 1(a) and 1(b)). It should also be noted that the hBMSCs from aged individuals (58, 59, and 65 years old) also required much longer time (20 days) to approach a similar cell culture density to those from the younger women. Since PEMF had an effect on preosteoblastic cell number from the younger women and cell proliferation involves activation of intracellular signaling pathways, especially extracellular regulated kinases (ERKs), we determined activation of these enzymes by PEMF. As shown in Figure 2(a), PEMF increased ERK activation (phosphorylation) after 15 min on day 5 in BMSCs from a 24-year-old woman. A similar effect was also found in hBMSCs from other younger female subjects (24- and 27-year-old women's cells) and the quantitative analysis of ERK activation (phosphorylated ERKs) from the three individuals after normalization to total ERKs confirmed the above result (Figure 2(b)). There was no significant activation of ERKs from any of these cells on the 10th day of PEMF treatment (Figures 2(c) and 2(d)).

**Figure 3:**
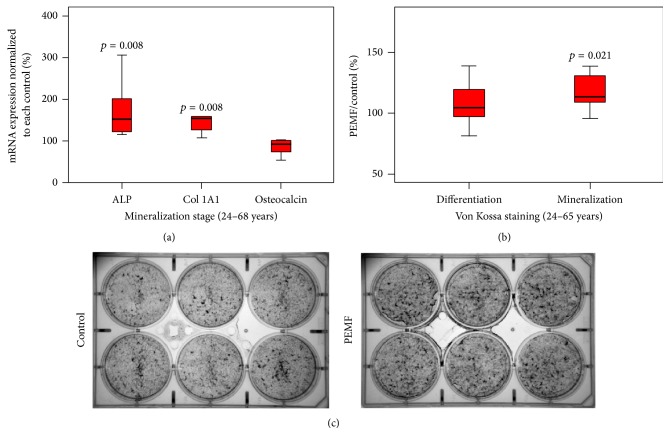
Effect of PEMF on expression of osteoblastic marker genes and mineralization in hBMSCs. Differentiating preosteoblasts from 24–68-year-old women were grown in the presence of osteoblast differentiation medium after confluence was reached and were treated with PEMF for 33 days or 43 days (59–68-year-old samples) of culture. (a) Total RNA was isolated and subjected to qRT-PCR using specific primers for human ALP, type I collagen, OC, and RPL13A. *n* = 9. (b) Cells were then subjected to Von Kossa staining and the mineralized calcium deposits were quantified. *n* = 9. Statistical analyses were conducted using Wilcoxon signed rank test. The *p* value ≤ 0.05 is considered as significant compared with the controls. (c) An example of Von Kossa staining and mineralized calcium deposits for hBMSCs of a 24-year-old female after 33 days of osteogenic culture in the presence or absence of daily Cervical-Stim PEMF.

To determine the role played by PEMF in osteoblast differentiation and mineralization of hBMSCs, experiments were carried out at molecular and cellular levels. At the molecular level, the mRNA expression of alkaline phosphatase (ALP), type I collagen (COL1A1), and osteocalcin (OC), which are known osteoblast differentiation and mineralization marker genes, was determined using qRT-PCR analysis. PEMF significantly increased mRNA expression of ALP and Col1 but not OC in BMSCs that had been allowed to proliferate, differentiate, and mineralize (Figure 3(a)). We next determined the effect of PEMF on mineralization in BMSCs by Von Kossa staining (Figures 3(b) and 3(c)). PEMF significantly stimulated mineralization of BMSCs in the mineralization phase and did not in the differentiation phase.

### 3.2. PEMF Regulation of Genes during hBMSC Proliferation by Microarray Analysis

A sample from a young individual was used for microarray analyses because PEMF significantly enhanced cell growth for young individuals compared to old individuals as shown in Figure 1. For assessment of the effect of PEMF on gene expression during hBMSC proliferation, on the 5th day of PEMF treatment 2 h after initiating the PEMF signal (pilot studies had shown significant PEMF stimulation of Cyclin gene expression at this time and day, data not shown), total RNA was isolated and used for the subsequent test with Affymetrix Human U133 plus 2.0 Gene Chips. After identifying significantly regulated genes, gene ontology analyses were performed by DAVID Bioinformatics Resources 6.7 software. The results indicated that PEMF stimulation of proliferating hBMSCs mainly affected genes of cell cycle regulation, cell structure, extracellular matrix (ECM), and some growth receptors or kinase pathways. There were a total of 114 known genes upregulated and 17 known genes downregulated at this time point (partially listed in Table 2). We have also included the decrease in fibrillin 2, even though it was not −1.5-fold, since this sequesters members of the TGF-*β* family and is the subject of our later research in this report.

### 3.3. PEMF Regulation of Genes in Differentiated and Mineralized hBMSCs by Microarray Analysis

In the differentiation (day 23) and mineralization stages (day 33) after daily 4 h PEMF treatment, a total of 37 (partially listed in Table 3) and 173 (partially listed in Table 4) known genes, respectively, were identified as significantly regulated. In these two stages, PEMF regulated preosteoblast gene expression and most genes were downregulated including transcriptional regulators, metabolism, proteases, and regulators and also cell adhesion and binding proteins and cytoskeletal and structural proteins. Changes in gene transcription of candidate genes chosen from microarray analyses were verified and confirmed by RT-qPCR on RNA from differentiated hBMSCs from 3 females, aged 24, 27, and 31 years (Table 5). Notably, the TGF-*β* signaling pathway seems to be most highly regulated by PEMF. In particular, RT-qPCR showed that fibrillin 2 (FBN2) was significantly decreased in expression by 65 ± 14%, while TGF-*β*2 mRNA significantly increased to 155 ± 44% and TGF-*β* regulator 1 (TBRG1) mRNA significantly increased to 143 ± 23%, relative to controls. In contrast, in mineralizing cells (Table 6), there was no decrease in FBN2 expression and a lesser significant increase in TGF-*β*2. It appears that PEMF stimulated a number of components of the TGF-*β* pathway in differentiating and mineralizing osteoblasts. It is notable that no components of the BMP pathway were seen to be regulated.

### 3.4. PEMF Activation of TGF-*β* Signaling via Smad2 in Differentiated and Mineralizing Osteoblasts

**Figure 4:**
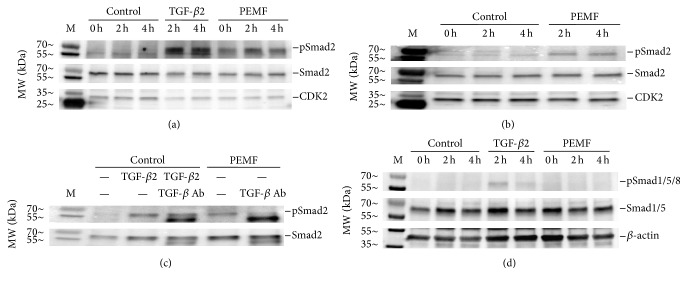
PEMF resulted in activation of the TGF-*β* signaling pathway in human osteoblastic cells during differentiation and mineralization. (a) Whole cell lysates after PEMF treatment of hBMSCs of a 24-year-old female at day 23 (differentiation) and (b) at day 33 (mineralization) were subjected to Western blot analysis using the antibodies as indicated for Smad2 and Cdk2. TGF-*β*2 (5 ng/mL) was added to control (non-PEMF-treated) cells on days 23 and 33 as positive controls. (c) The pan-TGF-*β* neutralizing antibody (30 ug/mL) was added to the osteogenic medium of hBMSCs from a 24-year-old female during the entire differentiation period and lysates were prepared on day 23 of PEMF treatment, 2 h after PEMF was started or TGF-*β*2 was added and subjected to Western blot analysis. TGF-*β*2 (5 ng/mL) was added to control (non-PEMF-treated) cells on day 23 as a positive control. Cdk2 was used as loading control. (d) The same lysates were subjected to Western blot analysis for phosphorylation of Smad1/5/8 as indicated.

To validate the PEMF effect on activation of the TGF-*β* signaling pathway, hBMSCs were subjected to differentiation (day 23) and mineralization (day 33) as described. During differentiation and mineralization, the cells were continuously treated with PEMF for 4 h each day. At days 23 and 33, cells were subjected to control, TGF-*β*2, or PEMF treatments for 0, 2, and 4 h. TGF-*β*2 was used as a positive control for activation of TGF-*β* signaling. Whole cell lysates were prepared and subjected to Western blot analyses using the antibodies for phosphorylated and total Smad2. The results show that PEMF stimulated activation of Smad2 by increased phosphorylation at day 23 in differentiated osteoblasts (Figure 4(a)) and less at day 33 in mineralizing osteoblasts (Figure 4(b)). To determine the specificity of activation of the TGF-*β* signaling by PEMF, osteoblasts were pretreated with pan-TGF-*β* antibody before PEMF treatment. The results show that the PEMF-stimulated Smad2 activation in differentiated osteoblasts (day 23) was blocked when cells were pretreated with pan-TGF-*β* antibody (Figure 4(c)). Since a recent paper has described a different PEMF signal as acting through the BMP pathway on rat calvarial osteoblasts [18], we examined whether Smad1/5/8 was phosphorylated in response to the Cervical-Stim signal in hBMSCs (Figure 4(d)). We were unable to observe any stimulation of this pathway, in contrast to the activation of the Smad2 pathway, even though the strong positive control, TGF-*β*2, slightly stimulated Smad1/5/8 phosphorylation, as has been observed by others [19, 20].

### 3.5. PEMF Stimulates Osteoblast Marker Gene Expression by Activation of the TGF-*β* Signaling Pathway

**Figure 5:**
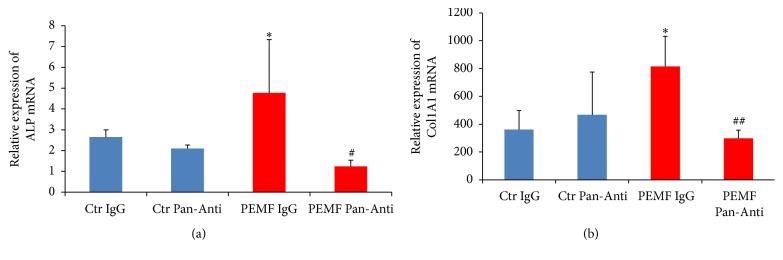
PEMF resulted in stimulation of expression of osteoblast differentiation marker genes via the TGF-*β* signaling pathway. Differentiated human osteoblasts derived from hBMSCs from a 30-year-old female were used. Total RNA was isolated after incubation with IgG or pan-TGF-*β* antibody (Pan-Anti) and treatment with control (Ctr) or PEMF and subjected to RT-qPCR using the primers for (a) ALP and (b) collagen 1A1 genes. RPL13 was used to normalize gene expression. *n* = 3. *∗* indicates significant increase compared with control IgG. # indicates significant decrease compared to all groups with ALP mRNA expression; ## indicates significant decrease compared to control or PEMF treatment with IgG incubation with collagen 1A1 mRNA expression; analysis by one-way ANOVA.

To determine if TGF-*β* signaling is responsible for the PEMF effect on expression of osteoblast differentiation marker genes such as ALP and type I collagen, this pathway was inhibited and RNA collected from differentiated osteoblasts at day 23 and subjected to RT-qPCR analysis. We found that PEMF significantly stimulated mRNA expression of ALP (Figure 5(a)) and type I collagen (Figure 5(b)) in differentiated osteoblasts. When cells were pretreated with pan-TGF-*β* antibody, PEMF stimulation of expression of these genes was significantly decreased (Figures 5(a) and 5(b)). Thus, this result indicates that the osteogenic effect of Cervical-Stim PEMF on hBMSCs is mediated via the TGF-*β* signaling pathway.

### 3.6. PEMF Stimulation of miR21-5p Expression in Differentiating Osteoblasts

**Figure 6:**
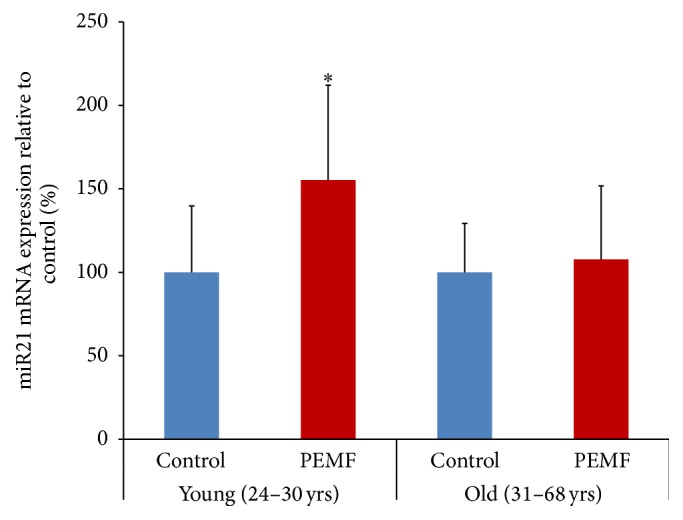
PEMF stimulated expression of miR21-5p in differentiated human osteoblasts. Total RNAs from control or PEMF-treated hBMSCs of females (24 × 2, 27, 29, and 30 years old, *n* = 5) at day 23 of differentiation or (31, 36, 58, and 68 years old) at day 23 or 33 of differentiation were isolated and subjected to RT-qPCR using the miScript II kit with miScript HiSpec Buffer and miScript SYBR Green PCR Kit. snoR10-1 was used to normalize miR21-5p expression and the expression is shown as a percentage of the relevant control samples. *∗* indicates significant increase compared to control using one-way ANOVA.

MicroRNAs are considered to be regulators of osteogenesis and bone formation. The microarray analysis of hBMSCs subjected to differentiation at day 23 identified the stimulation of expression of miR21 (Table 3). To verify this, total RNA was obtained with differentiated hBMSCs from females aged 24 × 2, 27, 29, and 30 (young individuals) and 31, 36, 58, and 68 (older individuals) years and subjected to RT-qPCR. The result shows that the expression of miR21-5p was 155% increased in cells from the younger women but not significantly increased in cells from the older individuals after PEMF treatment (Figure 6).

### 3.7. PEMF and miR21-5p Stimulation of Osteoblast Differentiation Marker Gene Expression

**Figure 7:**
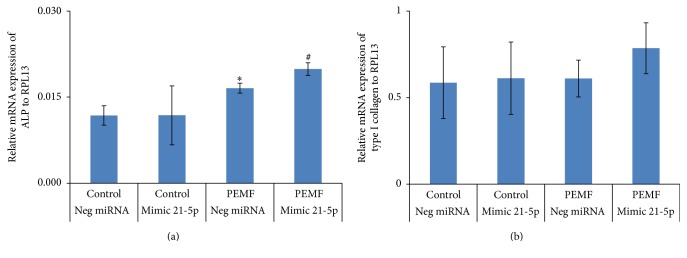
PEMF resulted in stimulation of expression of ALP mRNA and its effect was further enhanced by miR21-5p. Human BMSCs from a 31-year-old female were transiently transfected with 50 nM of negative control miRNA or miR-21-5p mimic for 72 h in osteogenic medium and PEMF treatment was carried out for 4 h each day for a total of 6 days. Total RNA was isolated and RT-qPCR was carried out using the primers for ALP (a) and collagen 1A1 (b) genes. Expression of the mRNAs is shown relative to the RPL13 gene. *n* = 3.  *∗* indicates significant increase compared to negative control miRNA transfection. # indicates significant increase compared to all treatments. Analysis by one-way ANOVA.

It is evident that PEMF stimulated miR21-5p expression in differentiated osteoblasts from younger individuals (Figure 6) which strongly suggested a role for miR21-5p in promotion of osteoblast differentiation. To determine this role, hBMSCs were transiently transfected with negative control miRNA or miR21-5p mimic for 3 days and concurrently subjected to 4 h PEMF treatment every day for 6 days. Total RNA was isolated and subjected to RT-qPCR analysis. When cells were treated with PEMF, there was significantly increased ALP mRNA expression. The miR21-5p mimic alone had no effect but together with PEMF treatment caused a significant increase in ALP mRNA expression compared with PEMF treatment alone (Figure 7(a)). With type I collagen mRNA expression, no significant effect was seen with respect to PEMF, miR21-5p mimic, or both treatments under these conditions (Figure 7(b)).

### 3.8. PEMF Regulation of Smad7 via miR21-5p in Differentiating Osteoblasts

**Figure 8:**
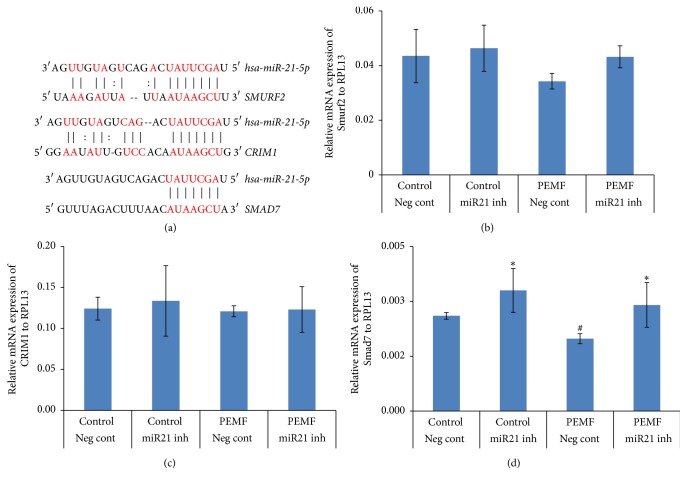
Putative target genes of miR21-5p and PEMF decreases Smad7 mRNA through miR21-5p. (a) The putative target region analysis was performed for Smurf2, Crim1, and Smad7 mRNAs 3′ UTR by miR21-5p seed sequence. ((b)–(d)) Human BMSCs from a 27-year-old female were transiently transfected with 50 nM of negative control miRNA or miR21-5p inhibitor for 72 h in osteogenic medium and PEMF treatment was carried out concurrently for 4 h each day for 3 days. Total RNA was isolated and RT-qPCR was carried out using the primers for (a) Smurf2, (b) Crim1, and (c) Smad7 genes. Expression of mRNAs is shown relative to that of the RPL13 gene. *n* = 3. *∗* indicates significant increase compared to negative control miRNA transfection or PEMF treatment with negative control miRNA transfection. # indicates significant decrease compared to PEMF treatment with miR21-5p inhibitor transfection. Analysis by one-way ANOVA.

In silico analysis (http://www.microrna.org/microrna/home.do) was used to identify the putative target genes of miR21-5p for its functional importance towards osteogenic commitment. Among them some antagonistic effectors of osteogenesis such as Smad7, Smurf1, and Crim1 were found. The 3′UTR regions of Smad7, Smurf1, and Crim1 held at least 6-nt perfect complementarities to the miR21-5p seed region (Figure 8(a)). To validate these putative target genes of miR21-5p, hBMSCs were transiently transfected with either negative control miRNA or miR21-5p inhibitor and concurrently treated with PEMF for 4 h each day for 3 days. To determine the expression level of these target genes, total RNA was isolated, followed by RT-qPCR analysis. There was no significant change in mRNA expression of Smurf2 (Figure 8(b)) and Crim1 (Figure 8(c)) in the cells in the presence of PEMF treatment, miR21-5p inhibitor, or both. In the case of Smad7, there was a significant decrease in its mRNA expression after PEMF treatment, and inclusion of miR21-5p inhibitor reversed the PEMF effect resulting in increased Smad7 mRNA expression (Figure 8(d)). From these results we suggest that Smad7, an antagonist of TGF-*β* signaling, is likely to be miR21-5p's target gene and PEMF downregulates its mRNA expression via miR21-5p in differentiating osteoblasts. In fact, at least two groups have shown that the 3′-UTR of Smad7 is, indeed, a target for miR21-5p, resulting in a decrease in Smad7 protein levels [21, 22].

### 3.9. PEMF Regulation of Runx2 Expression via miR21-5p and Smad7 in Differentiating Osteoblasts

**Figure 9:**
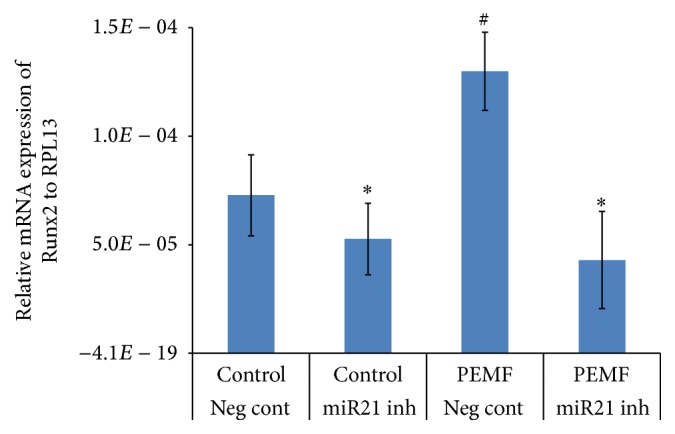
PEMF stimulated Runx2 expression and its effect was downregulated by miR21-5p inhibitor. Human BMSCs from a 27-year-old female were transiently transfected with 50 nM of negative control miRNA or miR21-5p inhibitor for 3 days in osteogenic medium and PEMF treatment was carried out concurrently for 4 h each day for 3 days. Total RNA was isolated and RT-qPCR was carried out using the primers for Runx2. Expression of Runx2 mRNA is shown relative to that of the RPL13 gene. *n* = 3. *∗* indicates significant decrease compared to negative control miRNA transfection or PEMF treatment with negative control miRNA transfection. # indicates significant increase compared to control treatment with negative control miRNA transfection. Analysis by one-way ANOVA.

**Figure 10:**
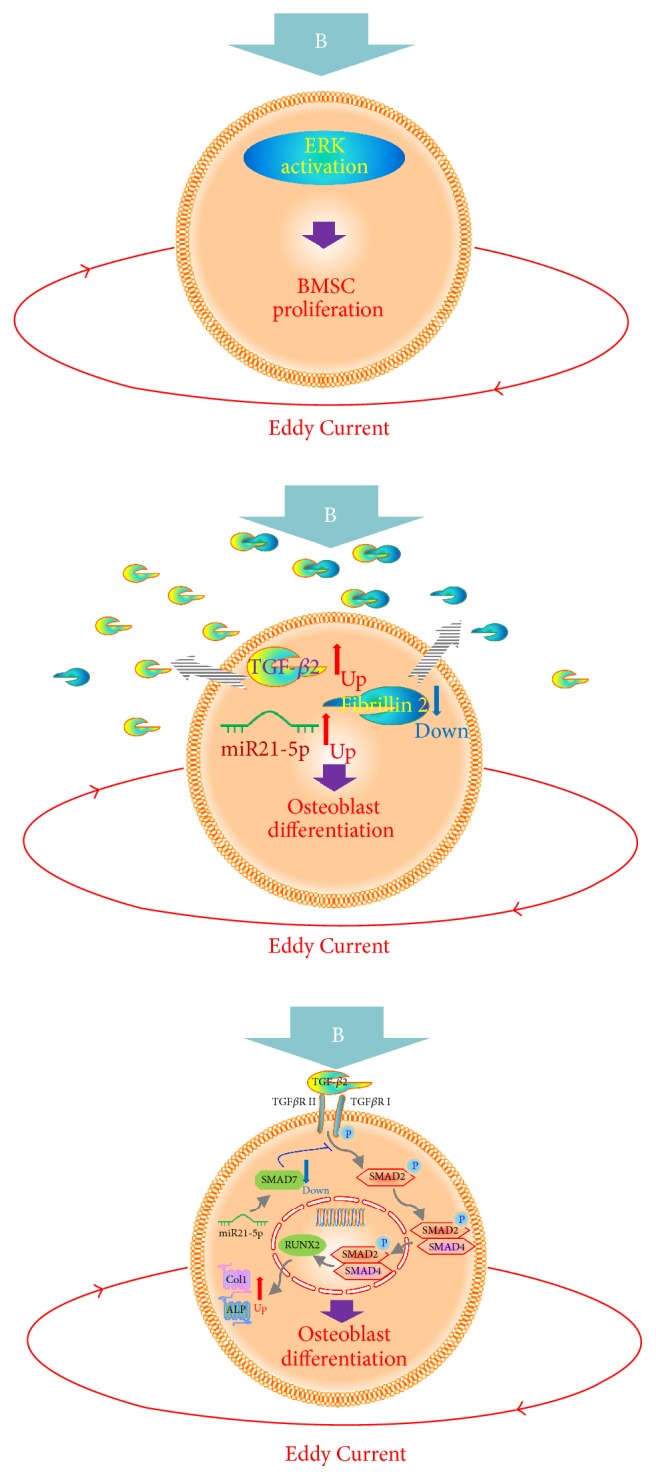
Schema of the mechanisms involved in PEMF stimulation of BMSC proliferation and osteoblast differentiation. The magnetic field (B) is thought to elicit Eddy Currents that act on BMSCs and cause activation of ERKs that are then involved in increased BMSC proliferation. After the BMSCs reach confluence and they are switched to differentiation medium, the magnetic field (B) and the resultant Eddy Currents cause a decrease in fibrillin 2 expression and an increase in TGF-*β*2 and miR21-5p expression. The decrease in fibrillin 2 would lead to an increase in the amount of available TGF-*β*2. The increase in miR21-5p appears to cause a decrease in inhibitory Smad7 expression, thus, enhancing TGF-*β*2 activation of Smad2 with resulting increase in Runx2, collagen I, and alkaline phosphatase expression in the cultures, that is, increased osteoblast differentiation.

Since Runx2 is required for osteoblast differentiation and PEMF stimulated expression of osteoblast differentiation marker genes (Figure 3), we next examined the PEMF stimulation of expression of Runx2 in differentiating hBMSCs and the role played by miR21-5p. Human BMSCs were transiently transfected with either negative control miRNA or miR-21-5p inhibitor, followed by PEMF treatment. Total RNA was isolated and subjected to RT-qPCR analysis. The result showed that there was a significant increase in expression of Runx2 mRNA in response to PEMF treatment and this effect was blocked by miR21-5p inhibitor in differentiating osteoblasts (Figure 9). From these results, we suggest that PEMF promotes its osteogenic effect via stimulation of miR21-5p expression and activation of TGF-*β* signaling in hBMSCs. A figure summarizing that the mechanisms we conclude are involved in PEMF stimulation of BMSCs and osteoblast differentiation is shown in Figure 10.

## 4. Discussion

Numerous studies have shown that mechanical stimulation of bone progenitors including ultrasound [23], mechanical strain [24, 25], and compression as well as shear forces has a stimulatory effect on bone progenitors involved in bone healing of critical size defects and nonunions in vivo. A broad set of investigations has aimed to unravel potential underlying molecular mechanisms and growth factor pathways involved with sophisticated in vitro methods [26]. A number of mechanisms have been proposed by which mechanical cues on different physical scales and identities can incorporate into growth factor signaling [27]. In particular, the major TGF-*β* growth factor superfamily of ligands (including TGF-*β* 1 and 2 as well as BMPs) and their downstream signaling via Smad2/3 and Smad1/5/8 transcription factors, respectively [28, 29], appears to be affected by mechanical stimulation in a diverse set of cells, with the majority of research focussing on bone progenitors, for example, BMSCs, osteoblasts, osteocytes, and chondrocytes. This is a large and ongoing field of study.

The molecular mechanisms responsible for the effect of PEMF on bone formation [14, 30] have not been completely elucidated. We found that PEMF promoted preosteoblast proliferation from hBMSCs from individuals up to age 30, but not older individuals, and stimulated differentiation marker gene expression of mineralizing hBMSCs of all ages. To dissect the mechanisms, PEMF effects on proliferation, differentiation, and mineralization of hBMSCs were examined by Affymetrix microarray analyses. We found that PEMF stimulation of hBMSC proliferation mainly affected genes of cell cycle regulation, cell structure, ECM, and some growth receptors or kinase pathways (Table 2). At the cellular and molecular levels, PEMF has been reported to promote the synthesis of ECM proteins and exert a direct effect on the production of proteins that regulate gene transcription. PEMF may affect several membrane receptors and stimulate osteoblasts to secrete several growth factors such as BMP-2 and BMP-4 and TGF-*β*. PEMF has been reported to affect osteoblast cellular proliferation and differentiation of bone cells in vitro by enhancing DNA synthesis [14, 31], increasing the expression of bone marker genes during differentiation and mineralization [7], and enhancing calcified matrix production. Several experimental studies also demonstrated that PEMF stimulation could potently promote osteogenesis and enhance bone mineralization both in vivo and in vitro [32–34].

The microarray data for PEMF regulation of differentiation and mineralization of hBMSCs showed regulation of transcriptional regulators, metabolism, proteases, cytokines and growth factors, and also cell adhesion and binding proteins and cytoskeletal and structural proteins (Tables 3 and 4). Identifying the signaling pathways and their associated regulatory mechanisms of PEMF action on osteogenesis might further promote its use in clinical applications. Thus, PEMF regulated preosteoblast gene expression during the differentiation and mineralization stages, and candidate genes chosen from microarray analyses were confirmed by RT-qPCR (Tables 5 and 6). Notably, the TGF-*β* signaling pathway and miR21 seem to be most highly regulated by PEMF. Thus, in the present study, we systematically investigated the mechanism of action of PEMF effects on osteogenesis via activation of TGF-*β* signaling and miR21-5p expression using hBMSCs.

The TGF-*β*/BMP signaling pathway plays a fundamental role in the regulation of bone organogenesis through the activation of receptor serine/threonine kinases. Perturbations of TGF-*β*/BMP activity are almost invariably linked to a wide variety of clinical outcomes including skeletal anomalies [28]. Phosphorylation of TGF-*β* (I/II) or BMP receptors activates intracellular downstream Smads, the transducer of TGF-*β*/BMP signals. In our studies, PEMF (Cervical-Stim) treatment activated only the Smad2 signaling component in differentiated hBMSCs (Figure 4) and activation of this signaling pathway appeared to be essential for PEMF stimulation of early osteoblast differentiation marker genes such as ALP and type I collagen (Figure 5). It is notable that it did not appear to activate the BMP pathway through Smad1/5/8 phosphorylation. The TGF-*β*/BMP signaling effect may be complex and highly time- and space-specific during skeletal development and bone formation. Very recently, Xie et al. [18] have described a different PEMF signal as operating through the BMP receptor on the primary cilium of rat calvarial osteoblasts in culture. Our accumulated data do not indicate that the BMP pathway is involved in the signaling mechanism of either Spinal-Stim or Cervical-Stim but we cannot rule out that it may have a role if investigated further. This signaling cascade can be modulated by various factors and other pathways [35, 36]. Activation of Wnt/Lrp5/*β*-catenin or calcium-related mechanisms by PEMF treatment for osteogenic activity have also been reported [37, 38].

Osteoblast differentiation is tightly controlled by several regulators including miRNAs [17, 39, 40] that can regulate expression of genes during differentiation of MSCs towards osteoblasts, resulting in the osteogenic lineage. Differential expression of miRNAs could be responsible for activation of several signaling pathways such as TGF-*β*/BMP, Wnt/*β*-catenin, and transcription factors [41]. PEMF stimulated miR21-5p expression in differentiated hBMSCs from younger females (Figure 6) suggesting one of the ways PEMF mediates its osteogenic effect on these cells is via miR21-5p. MicroRNA 21 was one of the first miRNAs detected in the human genome and it was found to be overexpressed in several types of cancer tissues [42]. A role for miR21 in cell proliferation and apoptosis has been reported [43]. With regard to the regulation of bone formation, a number of miRNAs are expressed in the developing skeletal system and miRNA-dependent modulation of gene function can alter skeletal phenotypes across individuals and also within the same individual over time [44]. MicroRNAs might have direct or indirect effects for their regulatory functions in osteoblast differentiation.

To study the functional role of miR21-5p during osteoblast differentiation by PEMF treatment, it was necessary to alter its endogenous expression/activity. Overexpression of miR21-5p (mimic) in differentiated hBMSCs had no effect on mRNA expression of ALP and type I collagen (Figure 7) but required PEMF to have an enhanced effect on ALP mRNA expression which suggests that PEMF could also involve other pathways and molecules in addition to miR21-5p for its osteogenic effects in these cells. The putative targets of miR21-5p can be classified according to their negative contribution in osteogenic differentiation or positive contribution to other lineages using online software. Among them are some key regulators or antagonistic effectors of osteogenesis such as Smad7, Smurf2, and Crim1 and these genes are well documented in their antagonistic roles in osteogenesis [29, 45]. Expression of the putative target genes in the presence of the miR21-5p inhibitor showed a significant increase in Smad7 mRNA expression in differentiated hBMSCs (Figure 8). The inhibitory Smads (Smad6, Smad7) potentially act as suppressors of bone formation. While Smad7 inhibits TGF-*β*/BMP signaling, Smad6 is less effective in inhibiting TGF-*β* signaling. It has been reported that Smad7 can inhibit ALP activity and suppress type I collagen mRNA and protein levels [46]. MicroRNA 21 has been shown to be a key regulator of TGF-*β* signaling [47] and Smad7 was found to be one of its target genes [21, 40, 43]. Other target genes such as PTEN and STAT3 have also been reported for miR21 [48, 49]. Based on our results (Figures 7 and 8), we suggest that Smad7 is a target gene for miR21-5p during PEMF regulation of osteoblast differentiation.

Since PEMF stimulates miR21-5p expression in differentiated hBMSCs (Figure 6) and miR21-5p targets Smad7 (Figure 8(d)), the PEMF action on osteogenesis via miR21-5p and Smad7 was further investigated. Runx2 is essential for the commitment of multipotent mesenchymal cells to the osteoblastic lineage. In general, Runx2 activity can be altered by its interacting proteins and/or posttranslational modifications [17, 50–54]. The steady-state protein level of Runx2 can be regulated by E3 ubiquitin ligases, Smurf1 and Smurf2, and it has been reported that the degradation of endogenous Runx2 can be blocked by a proteasomal inhibitor or by Smurf2 siRNA [55]. PEMF stimulated Runx2 mRNA in differentiated hBMSCs, and miR21-5p inhibitor prevented the PEMF stimulation of Runx2 expression (Figure 9). It has already been reported that Smad7 interacts with Smurf2 but it does not interact with Runx2 [56]. Hence, targeting Smad7 through miR21-5p by PEMF could possibly decrease the Smad7-dependent Smurf2 activity, resulting in stabilization of Runx2 protein, and feedback to increased transcription of Runx2 in differentiated hBMSCs. A figure summarizing that the mechanisms we conclude are involved in PEMF stimulation of BMSCs and osteoblast differentiation is shown in Figure 10. We can only speculate as to how PEMF regulates miR21-5p, but others have shown that this microRNA is regulated by transcriptional mechanisms, such as by myocardin-related transcription factor-A (58) or by STAT3 (59), and such mechanisms could possibly be implicated in PEMF's actions.

## 5. Conclusions

Our results show that PEMF significantly stimulated the cell number of preosteoblasts from BMSCs of young women while not stimulating those from women older than 30. We also showed that PEMF regulates a range of genes in hBMSCs to stimulate their proliferation, differentiation, and mineralization. Our further investigation suggests a novel regulatory mechanism of PEMF action during differentiation and mineralization of hBMSCs by activation of the TGF-*β* signaling pathway. PEMF appears to activate this pathway in hBMSCs of younger women by inhibiting Smad7 expression through miR21-5p and in turn PEMF controls the function of Runx2 resulting in promotion of its osteogenic effect.

## Figures and Tables

**Table 1:** Primers used in this study.

Gene name	Forward primer (5′ > 3′)	Reverse primer (5′ > 3′)
ALP	TGGACGGCCTGGACCTCGTT	AGGGTCAGGAGTTCCGTGCG
COL1A1	GGAGGCACGCGGAGTGTGAG	CCTCTTGGCCGTGCGTCAGG
Osteocalcin	GAGCCCCAGTCCCCTACCCG	GACACCCTAGACCGGGCCGT
FOSB	GCGCCGGGAACGAAATAAAC	TTCGTAGGGGATCTTGCAGC
LEPR	GTGGGGCTATTGGACTGACT	CTTTGAGAGTCCAGCAGGCA
TBRG1	GCTAGATTCCTAGAGGCCCG	GGCATCGGATCCTAAGTCGG
FBN2	CTTTAGGCCGGTTATGCAACG	AATAAGCCCTTCGTCGGCTC
SOX11	TTGGAAGCGGAGAGCAACCT	TGCGTTCGATCTTGGACCAT
CTNNA1	GGCAGCCAAAAGACAACAGG	GGCCTTATAGGCTGCGACAT
AKT3	CTCTATTATTTGGGCTGAGTCATCA	CCCCTCTTCTGAACCCAACC
CXCL12	GACAAGTGTGCATTGACCCG	TGTAAGGGTTCCTCAGGCGT
THBS1	CCTCTACTCCGGACGCAC	GCCCCGGTGAGTTCAAAGAT
COL5A1	CGGGGACTATGACTACGTGC	CTCCAAGTCATCCGCACCTT
GPC4	CAGAGGTCCAGGTTGACACC	TCGGCTTTCTCATTGGCACT
MMP16	TGCGGAACGGAGCAGTATTT	TGTGCTTGTGCTGCCATTTC
TGFB2	CCCCGGAGGTGATTTCCATC	AACTGGGCAGACAGTTTCGG
CDH11	CCCAGTACACGTTGATGGCT	ACGTTCCCACATTGGACCTC
SPP1	GCCTCCTAGGCATCACCTG	CTTACTTGGAAGGGTCTGTGGG
IL8	GGTGCAGTTTTGCCAAGGAG	TTCCTTGGGGTCCAGACAGA
RPL13A	AAGTACCAGGCAGTGACAG	CCTGTTTCCGTAGCCTCATG
hsa-miR-21-5p	UAGCUUAUCAGACUGAUGUUGA	

**Table 2:** Genes regulated by PEMF during hBMSCs proliferation by microarray analysis. Cells were from a normal 27-year-old female. Total RNA was isolated at day 5 after 2 h of PEMF treatment and used for microarray assays as described in Materials and Methods. Analysis by Student's *t*-test.

Gene symbol	Gene title	Fold-change (Avg PEMF versus avg controls)	*p*
*Cell adhesion and binding and cytoskeletal and structural proteins*			
MMP1	Matrix metallopeptidase 1 (interstitial collagenase)	4.57	2.16*E* − 03
PRC1	Protein regulator of cytokinesis 1	3.22	4.34*E* − 04
CCBE1	Collagen and calcium binding EGF domains 1	2.97	1.32*E* − 04
CLDN1	Claudin 1	2.71	2.26*E* − 04
CENPK	Centromere protein K	2.40	5.36*E* − 03
GAS2L3	Growth arrest-specific 2 like 3	2.39	4.74*E* − 03
CLDN11	Claudin 11	2.31	5.59*E* − 04
NUSAP1	Nucleolar and spindle associated protein 1	2.16	5.04*E* − 03
COL15A1	Collagen, type XV, alpha 1	2.12	1.64*E* − 04
HAPLN1	Hyaluronan and proteoglycan link protein 1	2.03	1.37*E* − 04
IBSP	Integrin-binding sialoprotein	1.97	2.17*E* − 03
FBN2	Fibrillin 2	−1.12	1.98*E* − 02
COL14A1	Collagen, type XIV, alpha 1	−2.30	1.56*E* − 02
MMP12	Matrix metallopeptidase 12 (macrophage elastase)	−3.27	1.19*E* − 04
MGP	Matrix Gla protein	−3.98	2.80*E* − 06

*p53 signaling pathway, apoptosis, and survival antiapoptotic TNFs/NF-kB/IAP pathway*			
BIRC5	Baculoviral IAP repeat containing 5	3.30	3.60*E* − 06
GTSE1	G-2 and S-phase expressed 1	2.48	1.02*E* − 03
SESN3	sestrin 3	−2.47	3.72*E* − 07

*Cell cycle role of APC (anaphase-promoting complex) in cell cycle regulation, cell cycle/checkpoint control*			
CDK1	Cyclin-dependent kinase 1	5.07	1.01*E* − 03
CDC20	Cell division cycle 20 homolog (*S. cerevisiae*)	3.00	3.32*E* − 04
CCNB2	Cyclin B2	2.41	1.83*E* − 03
NDC80	NDC80 kinetochore complex component homolog (*S. cerevisiae*)	2.38	7.59*E* − 04
TYMS	Thymidylate synthetase	2.36	9.43*E* − 05
CCNB1	Cyclin B1	2.35	2.17*E* − 03
NEK2	NIMA- (never in mitosis gene a-) related kinase 2	2.12	2.19*E* − 02
CCNA2	Cyclin A2	1.95	6.42*E* − 03
TTK	TTK protein kinase	1.95	1.77*E* − 02

*Akt signaling*			
CCL2	Chemokine (C-C motif) ligand 2	2.83	6.76*E* − 05
CSF2RB	Colony stimulating factor 2 receptor, beta, low-affinity	2.31	2.04*E* − 03

*Other receptor, kinase, and regulator*			
CDKN3	Cyclin-dependent kinase inhibitor 3	2.45	2.32*E* − 04
PDGFRA	Platelet-derived growth factor receptor, alpha polypeptide	2.26	2.52*E* − 05
CTSC	Cathepsin C	2.14	5.32*E* − 04
LEPR	Leptin receptor	−2.19	1.46*E* − 04
FGFR2	Fibroblast growth factor receptor 2	−2.04	8.83*E* − 05

**Table 3:** Genes regulated by PEMF in differentiating hBMSCs. Cells were from a normal 27-year-old female. Total RNA was isolated at day 23 of PEMF treatment. Analysis by Student's *t-*test.

Gene symbol	Gene title	Fold-change (Avg PEMF versus avg controls)	*p*
*Transcriptional regulator, RNA metabolism, and RNA transport*			
SPEN	Spen homolog, transcriptional regulator (*Drosophila*)	−1.74	2.68*E* − 02
FOXO3, FOXO3B	Forkhead box O3; forkhead box O3B pseudogene	−1.87	3.04*E* − 02
MIR21	MicroRNA 21	1.61	2.92*E* − 02

*Metabolic process *			
AKT3	v-akt murine thymoma viral oncogene homolog 3	−1.58	2.39*E* − 02

*Growth factor and regulator*			
TBRG1	Transforming growth factor beta regulator 1	1.72	9.85*E* − 03

*Receptor*			
LEPR	Leptin receptor	1.55	5.20*E* − 03

*Cell adhesion, motility, and cytoskeletal*			
ARPC5	Actin related protein 2/3 complex, subunit 5, 16 kDa	1.50	1.93*E* − 02
FBN2	Fibrillin 2	−1.45	1.47*E* − 02

*Signaling transduction, pathway*			
THBS1	Thrombospondin 1	−1.24	1.33*E* − 02

**Table 4:** Genes regulated by PEMF in mineralizing hBMSCs. Cells were from a normal 27-year-old female. Total RNA was isolated at day 33 of PEMF treatment. Analysis by Student's *t-*test.

Gene symbol	Gene title	Fold-change (Avg PEMF versus avg controls)	*p*
*Cell adhesion, motility, and cytoskeletal*			
COL1A2	Collagen, type I, alpha 2	−1.60	1.81*E* − 02
COL3A1	Collagen, type III, alpha 1	−1.61	9.49*E* − 03
FN1	Fibronectin 1	−1.93	2.31*E* − 04
FBN2	Fibrillin 2	1.38	2.49*E* − 02
VIM	Vimentin	−1.67	1.39*E* − 02

*Transcriptional regulator, RNA metabolism, and RNA transport*			
MIR21	MicroRNA 21	−2.16	1.28*E* − 02
HNRNPA1 LOC728844	Heterogeneous nuclear ribonucleoprotein	−1.91	1.41*E* − 02

*Cell cycle, cell growth, and apoptosis*			
CCNL1	Cyclin L1	−1.79	1.65*E* − 03
CCNL2	Cyclin L2	−2.07	3.18*E* − 03

*Hormone, growth factor, and cytokine*			
CXCL12	Chemokine (CXC motif) ligand 12 stromal cell-derived factor 1	1.60	3.83*E* − 03
IL15	Interleukin 15	−1.55	4.70*E* − 03
IL8	Interleukin 8	−2.00	3.71*E* − 02
TBRG1	Transforming growth factor beta regulator 1	−2.35	8.96*E* − 03
TGFB2	Transforming growth factor, beta 2	1.39	2.75*E* − 02

*Metabolic process*			
INSIG1	Insulin induced gene 1	1.52	2.36*E* − 02

*Signaling transduction, pathway*			
DAB2	Disabled homolog 2, mitogen-responsive phosphoprotein (*Drosophila*)	−1.62	3.65*E* − 02
THBS1	thrombospondin 1	1.52	3.67*E* − 03
TIFA	TRAF-interacting protein with forkhead-associated domain	−1.51	3.02*E* − 03

*Protease and regulator*			
SERPINE1	Serpin peptidase inhibitor, clade E member 1	−2.04	2.81*E* − 03
BAG2	BCL2-associated athanogene 2	−1.61	1.14*E* − 03

**Table 5:** Real-time RT-PCR of three different female donor samples' hBMSCs, aged 24, 27, and 31 years in the differentiation stage. Analysis by Student's *t-*test.

Gene symbol	Gene title	Average PEMF/control %	*p*
COL1A1	Collagen, type I, alpha 1	133 ± 24%	3.90*E* − 02
COL5A1	Collagen, type V, alpha 1	136 ± 25%	3.44*E* − 02
CTNNA1	Catenin (cadherin-associated protein), alpha 1, 102 kDa	124 ± 3%	8.27*E* − 05
FOSB	FBJ murine osteosarcoma viral oncogene homolog B	127 ± 33%	1.11*E* − 01
SOX11	SRY- (sex determining region Y-) box 11	138 ± 24%	2.59*E* − 02
SPP1	Secreted phosphoprotein 1	131 ± 41%	1.31*E* − 01
TGFB2	Transforming growth factor, beta 2	155 ± 44%	4.87*E* − 02
TBRG1	Transforming growth factor beta regulator 1	143 ± 23%	1.65*E* − 02
AKT3	v-akt murine thymoma viral oncogene homolog 3 (protein kinase B, gamma)	74 ± 15%	2.06*E* − 02
FBN2	Fibrillin 2	35 ± 14%	7.38*E* − 04
IL8	Interleukin 8	56 ± 35%	4.87*E* − 02

**Table 6:** Real-time RT-PCR analysis of three different female donor samples' hBMSCs, aged 24, 27, and 31 years in the mineralization stage. Analysis by Student's *t*-test.

Gene symbol	Gene title	Average PEMF/control %	*p*
CDH11	Cadherin 11, type 2, OB-cadherin (osteoblast)	130 ± 36%	1.10*E* − 01
COL1A1	Collagen, type I, alpha 1	144 ± 47%	9.20*E* − 02
CXCL12	Chemokine (C-X-C motif) ligand 12	146 ± 20%	8.48*E* − 03
FBN2	Fibrillin 2	149 ± 54%	9.82*E* − 02
FOSB	FBJ murine osteosarcoma viral oncogene homolog B	165 ± 27%	6.97*E* − 03
GPC4	Glypican 4	128 ± 25%	6.23*E* − 02
IL8	Interleukin 8	162 ± 68%	9.49*E* − 02
LEPR	Leptin receptor	130 ± 19%	2.53*E* − 02
MMP16	Matrix metallopeptidase 16 (membrane-inserted)	135 ± 68%	2.07*E* − 01
SOX11	SRY- (sex determining region Y-) box 11	137 ± 37%	7.60*E* − 02
SPP1	Secreted phosphoprotein 1	132 ± 52%	1.74*E* − 01
TGFB2	Transforming growth factor, beta 2	128 ± 21%	3.99*E* − 02
TBRG1	Transforming growth factor beta regulator 1	113 ± 14%	9.71*E* − 02
THBS1	Thrombospondin 1	142 ± 58%	1.37*E* − 01
